# To Abortionists

**Published:** 1873-07

**Authors:** 


**_To Abortionists,_**

Who occasionally favor us with their confidential communications, expressing their deep interest in "suffering womanhood," their sympathy with the "tender sex" in the "dangers of maternity," their anxiety to promote the "mental, physical and moral (!) well-being of humanity," with a great deal more of the same disgusting bosh, and finally, and most important of all, their entire willingness to aid, abet, or commit murder, *i. e.*, fœticide, by pastille, bougie, sound, syringe, or in whatever way may be most secret, safe and profitable!—we have to say, that we are now prepared to give their circulars a fitting reception, and to dispose of them in a manner most conducive to the protection of womanhood and the promotion of the best interests of mankind. We shall therefore be happy to hear from any and all of these philanthropists! and to introduce their communications to the notice of the United States District Attorney, for indictment under the recent Act of Congress, prohibiting the transmission of obscene publications through the United States Mails.

The "Act" referred to is comprehensive in its scope, and specifies, as objects within its meaning, all statements having reference to the procuring of abortions, or to the *prevention of conception.*

The last communication of this kind, which we have received, is now in the hands of the United States District Attorney for the Northern District of Illinois, a gentleman thoroughly appreciative of the true legal and social relations of this crime and its perpetrators, entirely aware of the extent of his own power in regard to them, and determined to use it to its utmost limits to accomplish the eradication of the most pestilential evil of the day, an evil whose enormity and universality is known to few, although its unrecognized effects are patent to all in the wide-spread depreciation in female health in the so-called upper classes of society, in the alarming diminution in the birth-rate, and in the general social demoralization.

We have watched with intense interest for many years, the ineffectual attempts to suppress this crime, by the punishment of its perpetrators after the fact, and have been driven to the conviction that the extreme difficulty, indeed almost utter impossibility, of convicting offenders was due not so much to the absence of evidence as to the lack of appreciation by public sentiment (of which juries are supposed to be the exponents) of the true nature of the crime. Until the public mind is impressed with the truth that fœticide is *murder*—not manslaughter, but *murder*—we will look in vain for the conviction of its perpetrators by juries of any graver offenses than midemeanors.

It is by the medical profession alone that this enlightenment of public sentiment must be inaugurated, and, with the co-operation of the secular press, be accomplished. The platform occupied by a majority of the clergy, at the present time, is too narrow and too insecure in its foundation to justify the expectation of very efficient aid from that quarter.

We recognize in the recent Act of Congress, a means which promises more than any other yet devised for the total eradication of this fashionable form of diabolism, as it strikes at its very source and origin rather than at its final consummation.

A noxious plant is not destroyed by the destruction of its fruit, but by cutting away it roots through which it derives its sustenance and support. If therefore, physicians, who have the interest not only of humanity but also of their own profession at heart, will be faithful and zealous in bringing to the notice of the proper authorities all publications of this character which may fall into their hands, they will not only aid most efficiently in protecting society at large from the greatest curse which now rests upon it, but will also remove from their own profession a foul excrescence which now deforms it. W. H.

